# The Effects of Intra-articular Platelet-Rich Plasma Injections in Rheumatoid Arthritis: A Narrative Review

**DOI:** 10.7759/cureus.28182

**Published:** 2022-08-19

**Authors:** Frederico Moeda, Xavier Melo, Madjer Hatia, Sérgio Pinho, Duarte Calado, Jaime C Branco, Maria J Gonçalves

**Affiliations:** 1 Physical Medicine and Rehabilitation, Centro Hospitalar de Lisboa Ocidental, Lisboa, PRT; 2 Rheumatology, Centro Hospitalar de Lisboa Ocidental, Lisboa, PRT

**Keywords:** platelet-rich plasma, rheumatoid arthritis, antigen-induced arthritis, synoviocytes, chondrogenesis

## Abstract

Platelet-rich plasma injections have been a therapeutic option with exponential growth in several pathologies in the last decades, particularly musculoskeletal for their effect on improving pain and functionality. Rheumatoid arthritis is a chronic joint disease, which involves inflammation of the synovial membrane with cartilage and juxta-articular bone destruction. Conventional and biological disease-modifying anti-rheumatoid drugs are the cornerstone of the treatment of this disease. However, the use of intra-articular glucocorticoids is often necessary and the role of platelet-rich plasma injections in these patients remains uncertain.

A literature review was carried out through the PubMed database, Cochrane and Google Scholar for the search terms “rheumatoid arthritis” and “platelet-rich plasma”. Eleven studies have been included in this review: two of these are *in vitro* studies, five are animal studies, one case report, two case series and one randomized controlled trial. Most of the studies demonstrated a decrease in pain and inflammatory mediators and improvement of functional outcomes, with no severe adverse effects reported.

However, the quantity and quality of literature about the effects and safety of plasma-rich plasma injections in rheumatoid arthritis patients are still scarce. It is essential that well-designed randomized controlled trials are made on this topic to understand if platelet-rich plasma may be useful as a coadjuvant therapy in rheumatoid arthritis.

## Introduction and background

Rheumatoid arthritis (RA) is an autoimmune disease, which is characterized by a symmetric, inflammatory and peripheral polyarthritis. It affects 0.5%-1% of adults, with 5-50 per 100,000 new cases annually in industrialised countries. Female patients are often more affected than male patients and its onset is usually between 30-60 years [1-3]. It is associated with other organ-specific manifestations in about 40% of the cases (such as subcutaneous nodules, Sjögren syndrome or rheumatoid vasculitis) and it is often related to severe and disabling diseases with worse outcomes in this group of patients [4].

The pathological processes in this autoimmune disorder are mediated by many cytokines, proteases, cell adhesion molecules and angiogenic factors that lead to activation of fibroblast-like synoviocytes (FLS) and progressive articular degeneration. In patients with RA, there is also a prolonged survival and resistance to apoptosis of FLS. Typically, it causes persistent inflammation in small joints with synovitis and joint erosions [5].

The main therapeutic agents in RA are conventional disease-modifying anti-rheumatoid drugs (DMARDs), like methotrexate (MTX), which may be combined with other drugs such as biological agents or glucocorticoids (oral or intra-articular). Scientific advances and more profound knowledge about RA pathophysiology allowed early treatment of this disease with the prevention of joint damage and less disability. However, some patients may fail treatment with these agents or have significant adverse effects that compromise adherence to therapy. After 10 years of active disease, there is a very significant number of patients with total knee or hip replacement surgery, unable to work and with substantial functional disability [6].

Platelet-rich plasma (PRP) is stipulated as plasma with a platelet concentration above baseline [7]. There is no established platelet concentration range for the definition of PRP but is reported to range from 300,000 to 1,000,000/µL [8]. The cytokines in platelet alpha granules provide a regenerative stimulus that increases healing and promotes repair in tissues with low healing potential. Platelets also stimulate cell recruitment, differentiation and communication by sustained release of growth factors. PRP can be further divided into leukocyte-rich PRP or leukocyte-poor PRP, based on its cellular composition. Leukocytes are essential mediators of the inflammatory response, host defense against infectious agents and wound healing. Neutrophils participate in the phase of inflammation and wound healing. Monocytes and macrophages facilitate tissue repair by debriding and phagocytizing damaged tissue and debris. Like platelets, macrophages also secrete growth factors that are important in tissue repair and have been shown to contribute to subchondral bone regeneration [9,10]. PRP injections cause modulation of an intra-articular environment which can promote chondrogenesis, reduce inflammation and have cell-proliferative and antinociceptive properties. It is believed that it stimulates the proliferation of epidermal growth factor (EGF), transforming growth factor β (TGF-β), insulin-like growth factor (IGF), vascular endothelial growth factor (VEGF) and inhibits the proliferation of tumour necrosis factor α (TNF-α) and matrix metalloproteinase (MMP) enzymes [11]. PRP preparations are commonly activated prior to administration to induce a highly concentrated bolus release of growth factors to the target tissue [12].

The most robust data supporting PRP injections is in lateral epicondylopathy [13-16], gluteus medius tendinopathy [17], plantar fasciopathy [18], and knee osteoarthritis [19], improving pain and functional outcomes. As it is an autologous product, PRP does not have risk for immunogenic reactions and no severe adverse effects have been reported [20,21].

In the literature, investigators have used a variety of protocols, differing in preparation kits, centrifugation systems, number of centrifugation steps, activation methods with or without thrombin and/or calcium and concentrations of components. The great variability in these formulations creates a challenge to draw precise conclusions and determine the indications for their use [22,23]. The aim of this study is to review the literature about the efficacy and safety of intra-articular PRP injections in patients with RA.

Methods

We have reviewed case studies, case series, original studies (in vitro, animal, observational studies and randomized controlled trials) and reviews indexed in PubMed, Cochrane Library and Google Scholar until July 2022 for the following search terms: “rheumatoid arthritis” and “platelet-rich plasma”. We selected all the articles that analysed the effects of PRP in RA, antigen-induced arthritis and in vitro cell lines mimetizing RA.

## Review

In vitro studies

Yan et al. demonstrated that PRP may promote migration and invasion of RA-FLS, induce an increase in the formation of filopodia and lamellipodia in RA-FLS and upregulate the levels of MMP-1. These changes have been associated with a critical step in the aggravation of RA and these findings raise the question of the safety of using PRP in these patients [24].

Tong et al. studied the effect of PRP on synovial fibroblasts stimulated by lipopolysaccharide (LPS) to simulate RA conditions. This study found that PRP could decrease the production of inflammatory mediators after LPS stimulation, in particular, interleukin (IL) 6, TNF-α and IL-1β. Furthermore, it was also shown that PRP decreased angiogenesis in vitro and reversed the up-regulation of cell viability induced by LPS in synovial fibroblasts, suggesting a beneficial effect of this treatment in RA [25].

The main purpose of this article is to review the effects of using PRP injections in RA patients. However, we included in vitro studies given the limited amount of information on this topic, the importance of knowing these cell lines' behaviour when exposed to PRP and its relationship with the pathophysiology of RA.

These articles show that PRP may promote the migration and invasion of RA-FLS, but, at the same time, it may also be important in the suppression of inflammation, leaving its global effect unknown. These results are also consistent with other published studies with PRP extracts such as platelet-releasing growth factor [26] and extracted platelet-derived microparticles [27].

Thus, given the scarcity of data on the subject and results that may be conflicting, it is necessary to be cautious in their interpretation and inference to clinical practice.

Animal studies

Lippross et al. aimed to study the effects of PRP intra-articular injection on six-month-old pigs with antigen-induced arthritis. Pigs received two intravenous and intra-articular bovine serum albumin (BSA) injections (0.8mL/kg and 5mg/mL, respectively) and, two and four weeks later, 5mL of PRP was injected into their knee joints. PRP-treated pigs, when compared to those which did not receive PRP injections, showed a decrease in synovial IL-6, IL-1, IGF-1 levels and chondral protein concentration was restored to pre-arthritic level [28].

Pacheco et al. studied the outcome of two intra-articular knee PRP injections of 100µl in rats with complete Freund’s adjuvant-induced arthritis. In these rats, histologically, it was reported a higher level of subchondral bone formation and lower granulation and necrotic tissue when compared to the control group [29].

Tong et al. described a marked reduction of synovial hyperplasia, inflammatory cell infiltration and destruction of cartilage in the joints of PRP-treated mice with type II collagen-induced arthritis when compared to the treatment with dexamethasone. They also stated a decrease in expression of IL-6, IL-8, MMP-3, IL-17, IL-1β, TNF-α, receptor activator of nuclear factor kappa-B (RANKL) and interferon (IFN)-γ in inflammatory tissue [30].

Shafik et al. demonstrated a decrease in the number of inflammatory cells and the levels of high mobility group box-1, beclin-1 and tissue myeloperoxidase activity after PRP intra-peritoneally injection (0.5mL/kg, two days/week, during eight weeks) in rats with type II collagen-induced arthritis. These levels were even lower in rats receiving PRP plus glycyrrhizin injection, suggesting an ability to inhibit autophagic and inflammatory pathways [31].

Finally, Naujokat et al. aimed to study the effect of PRP in temporomandibular joints (TMJ) after antigen-induced arthritis. Domestic pigs received intra-muscular and intra-articular BSA in TMJ, which caused arthritis, verified by histological analyses of the synovial membrane, cartilage, the underlying bone and protein analyses. Two intra-articular administrations of 5mL of PRP were administered once a week after the last BSA injection in TMJ. In PRP-treated joints, concentration levels of IL-1β and TNF-α, hyperplasia of the synovial membrane and leucocyte infiltration were significantly decreased [32].

All animal studies included in this review show a beneficial effect of using PRP in decreasing inflammatory cytokines in antigen-induced arthritis. These results should be analysed with prudence, since the sample size of each study is relatively small and, although quite useful for the development of new treatments such as cytokine blockade therapies, no models perfectly duplicate the condition of human RA. However, these results suggest very promising effects of the use of PRP in cases of arthritis.

Human studies

Case Report

Stumberga reported a case of a 45-year-old male patient suffering from an erosive seropositive RA for two years, treated with MTX 15mg once weekly. He presented with bilateral knee pain and a bilateral intra-articular PRP injection was administered. After two weeks, tenderness and swelling knees worsening were present. Blood tests showed leucocytosis and CPR above normal. MTX was raised to 20mg per week and methylprednisolone was added to 8mg per day. After one month, pain and swelling had slightly decreased but the patient still needed to use crutches. This case report highlights the necessity for more experience and evidence to consider this treatment and to understand if PRP injection can promote exacerbation of joint swelling and synovitis or if it is the expected evolution before improving [5].

Case Series

Badsha et al. presented a case series of leukocyte-poor PRP ultrasound-guided injections (2-4mL) in four patients with RA who had persistent pain in the knees or wrists and inadequate response to intra-articular corticosteroids. The four patients were all female with a median age of 40 years old and treated with a biological or conventional DMARD.

There were no adverse reactions described over the mean five-month follow-up period and all patients showed improvement on the visual analogue pain scale and disease activity score of 28 joints (DAS28) and some also improved the severity of synovitis on Grey-scale and Power Doppler ultrasound [33].

Shively et al. presented a case series of three case reports (two male and one female patient) in which 0.5mL intra-articular and 1.5mL peri-articular PRP injections were used for the treatment of RA in patients seeking pain control and improved range of motion in metacarpophalangeal or proximal interphalangeal joints. The patients had a median age of 60 years old. Over the course of six months after PRP injections, all three patients reported a 20%-50% decrease in pain and functional impact of their illness when compared to the initial visit.

There were no adverse effects observed during the six months follow-up. However, it is important to highlight that it is not known if any of the patients were taking any daily medication such as glucocorticoids [34].

Randomized Controlled Trial

To our knowledge, there is only one randomized controlled trial (RCT) that evaluates the use of PRP injections in RA patients. Saif et al. aimed to study the effect of intra-articular PRP versus corticosteroid in RA patients with regard to local joint inflammation, disease activity, serum inflammatory cytokines and quality of life. The 60 patients included in the trial met the 2010 ACR-EULAR classification criteria for RA and they all had low disease activity regarding DAS28 (between 2.6 and 3.2). Furthermore, they had been treated with non-biological DMARDs for at least three months and had persistent pain or swelling in one or two medium-sized joints (wrist, elbows or ankles). Patients with local or systemic infections, malignancies, medicated with opioids, moderate or high disease activity, blood disorders and with previous steroid or PRP injections (four weeks and six months respectively) were excluded.

Patients were divided into two groups: Group 1 which included 30 patients (46 joints) who received 3-4mL intra-articular PRP once a month for three months and Group 2 which included 30 patients (43 joints) who received one intra-articular injection of 40mg of triamcinolone acetonide.

There was a statistically significant improvement in the visual analogue pain scale, swelling, tenderness, health assessment questionnaire disability index, DAS28, serum IL-1β and TNF-α at three months post-treatment in both groups and this improvement was only persistent at six months in the PRP group. There was no report of adverse effects in patients of both groups during the six months of follow-up.

According to this RCT, intra-articular PRP may be safe, and effective and may have a long-lasting effect than triamcinolone acetonide in RA patients with low disease activity and persistent inflammation in one or two medium-sized joints. It is important to emphasize, however, that this was an open labelled study and that synovial hypertrophy before and after the treatment was not studied by ultrasound, which would have been useful [35]. In these human studies, there seems to be an improvement in clinical and functional outcomes in RA patients after PRP injections.

However, there is significant heterogeneity in the studies presented, namely in the type and number of joints analysed, amount of PRP, preparation method (leukocyte-rich versus leukocyte-poor PRP, activated or not) and administration method (blind versus ultrasound-guided). Furthermore, in some studies, there is no mention of some of these aspects. Moreover, to our knowledge, there is only one RCT published in this area. All of these factors together with the scarce information published on the subject constitute the major limitations of this review and reflect the complexity of this therapy and the challenges in translating clinical trial findings into clinical practice. Therefore, we cannot suggest a specific dose of administration nor whether single or multiple administrations are preferable.

Given the benefit of this therapeutic option in some musculoskeletal pathologies such as knee osteoarthritis, a well-designed analysis on this topic would be beneficial, especially because, in most studies, patients with knee osteoarthritis concomitantly with RA are often excluded. Furthermore, there are no reported serious adverse effects of its use, which could be an advantage over other therapies such as the use of intra-articular glucocorticoids with some risks with multiple administrations [36]. Another important aspect to discuss is that most RA patients take nonsteroidal anti-inflammatory drugs regularly, which can be a problem, as PRP may not achieve its therapeutic potential because platelet function and aggregation are impaired in these cases [37]. These effects may also occur with other drugs, such as pioglitazone [38].

## Conclusions

In the last years, new intra-articular treatments, such as PRP, have been studied for the treatment of musculoskeletal pathologies with a positive impact on pain relief, and functional outcomes and with evidence of decreasing inflammatory changes and accelerating the regeneration process. The use of PRP injections appears to have promising results in RA patients. In in vitro studies, there were conflicting observations. On the one hand, it was found that PRP could decrease the production of inflammatory mediators. On the other hand, it was observed that PRP may promote migration and invasion of RA-FLS. In animal studies, PRP injections may improve arthritic changes in the synovium and cartilage and decrease inflammatory mediators in antigen-induced arthritis. In human studies, there seems to be also an improvement in most clinical and functional outcomes in RA patients after PRP administration. No severe adverse effects have been reported in the literature.

However, there is still a lack of good quality scientific studies, mainly RCTs. Furthermore, in the different published studies, there are different methods of collection, preparation, administration, and frequency of administration of PRP injections, which affects the ability to draw reliable conclusions. Therefore, In conclusion, it is important that well-designed clinical trials are carried out, to clarify the role of PRP in these patients and whether it may be useful and safe as an additional therapy in RA.
